# Better Than I Thought: Positive Evaluation Bias in Hypomania

**DOI:** 10.1371/journal.pone.0047754

**Published:** 2012-10-17

**Authors:** Liam Mason, Noreen O'Sullivan, Richard P. Bentall, Wael El-Deredy

**Affiliations:** 1 School of Psychological Sciences, University of Manchester, Manchester, Lancashire, United Kingdom; 2 School of Health Sciences, University of Liverpool, Liverpool, Lancashire, United Kingdom; University of Barcelona, Spain

## Abstract

**Background:**

Mania is characterised by increased impulsivity and risk-taking, and psychological accounts argue that these features may be due to hypersensitivity to reward. The neurobiological mechanisms remain poorly understood. Here we examine reinforcement learning and sensitivity to both reward and punishment outcomes in hypomania-prone individuals not receiving pharmacotherapy.

**Method:**

We recorded EEG from 45 healthy individuals split into three groups by low, intermediate and high self-reported hypomanic traits. Participants played a computerised card game in which they learned the reward contingencies of three cues. Neural responses to monetary gain and loss were measured using the feedback-related negativity (FRN), a component implicated in motivational outcome evaluation and reinforcement learning.

**Results:**

As predicted, rewards elicited a smaller FRN in the hypomania-prone group relative to the low hypomania group, indicative of greater reward responsiveness. The hypomania-prone group also showed smaller FRN to losses, indicating diminished response to negative feedback.

**Conclusion:**

Our findings indicate that proneness to hypomania is associated with both reward hypersensitivity and discounting of punishment. This positive evaluation bias may be driven by aberrant reinforcement learning signals, which fail to update future expectations. This provides a possible neural mechanism explaining risk-taking and impaired reinforcement learning in BD. Further research will be needed to explore the potential value of the FRN as a biological vulnerability marker for mania and pathological risk-taking.

## Introduction

Bipolar Disorder (BD) is characterised by episodes of mania and depression, interspersed with periods of relatively normal functioning. Pervasive impairments in decision-making are present in all phases of the disorder [1]–[3], marked by increased goal-pursuit, impulsivity and risk-taking activities with high potential for damaging consequences in manic episodes (including substance use, unprotected sex, gambling and spending sprees; DSM-IV-TR, [4]). Psychological models are consistent with these features being due to increased sensitivity to rewarding events, and argue that increased activity in a Behavioural Approach System (BAS; [5]) produces concomitant increases in manic symptoms [6], [7]. Conversely, reduced BAS activation is linked to depressive symptoms such as apathy, anhedonia and amotivation (see [7] for discussion of the BAS dysregulation theory). In this way BD may be associated with dysregulation in the processing of rewarding outcomes. Factor analytic [8], cross-sectional [9], [10] and longitudinal [11] designs indicate that mania and depression are relatively independent phenomena in BD. This allows the underlying cognitive basis for mania to be explored separately from vulnerability to depression.

Clinical populations of mania are typically in receipt of psychotropic medication, and frequently experience hospitalisation and high rates of comorbidity, all of which present a challenge to studying psychological processes associated with BD. Manic symptoms are known to lie on a spectrum that extends into the general population [12], [13], making it possible to identify individuals in the general population experiencing attenuated symptoms. The Hypomanic Personality Scale (HPS) identifies people meeting criteria for bipolar spectrum disorder but not yet in treatment [14], and predicts clinical episodes after thirteen-year follow-up [15]. HPS also correlates with trait measures of reward sensitivity (the BIS/BAS scales; [16], [17]). Hence it is possible to study reward processing in populations exhibiting similar cognitive biases whilst avoiding confounds from psychotropic medication, hospitalisation and comorbidity.

Reward processing has been linked to mesocorticolimbic pathways projecting from midbrain structures to orbitofrontal and anterior cingulate cortices [18], with dopamine (DA) encoding both anticipation and experience of reward stimuli [19]. Abnormal DA neurotransmission is a hallmark feature of BD [20], [21], with DA-antagonists ameliorating manic episodes [22] and evidence that antidepressants may ultimately exert their therapeutic effect via the DA system (e.g. [23]). Experimentally, mania has been associated with aberrant reward-related activity in DA-rich midbrain structures [24], although confounds from medication cannot be completely ruled out. This is especially problematic given that pharmacological agents act on the neural circuitry that mediates reward processing, as illustrated, for example, in the finding of disrupted reward-related activity following single doses of an antipsychotic in healthy controls [25]. We have previously found that functional activity in striatum in response to rewarding outcomes was more strongly modulated by reward value in a hypomania sample [26]. Similar patterns of activity have been reported in clinical populations exhibiting impulse-control disorders [27] and in healthy individuals receiving L-DOPA, a dopamine precursor [25]. Event-related potentials (ERPs) offer greater temporal resolution to investigate reinforcement learning processes in (hypo)mania.

The feedback-related negativity (FRN) is an event-related component that occurs as a negative deflection (260–320 ms) and is implicated in motivational processing, appearing larger (*i.e*. more negative) for worse-than-expected outcomes and attenuated (more positive) or absent for better-than-expected outcomes (see [28]). In this way the FRN may represent a system subjectively evaluating outcomes along a good-bad continuum [29], which therefore makes it a useful tool for probing individual differences in sensitivity to reward and punishment outcomes. The FRN is also linked to learning of motivational outcomes, with an influential theory stating that its amplitude reflects a reversal of the prediction error signal (the difference between the predicted and actual outcome) generated in the midbrain [28], [30]. Experimental evidence generally demonstrates that the FRN conforms to associative learning theory assumptions [31], [32]. Therefore this component is also a useful tool for probing reward learning deficits, which have been previously implicated in clinical populations of BD [33].

While the FRN has not been investigated in relation to mania, depressive symptoms are associated with larger FRN (*i.e* a greater negative deflection), most notably for losses and negative feedback [34]–[36]. This is consistent with a hypersensitivity to adverse events and a bias towards negative (self-)evaluation. Further, there is evidence that the FRN elicited by positive feedback (e.g. monetary reward) is also larger in individuals exhibiting depressive symptoms (i.e. the FRN appears more loss-like; [37]). In this way depression is also characterised by blunted reward sensitivity, consistent with neuroimaging studies showing reduced reward-related activity in midbrain regions [38], [39]. Conversely, impulsivity is associated with a tendency to overvalue rewards [40] and a failure to learn from the negative consequences of behaviour [i.e. reduced punishment sensitivity; 41]. Consequently impulsive individuals exhibit the opposite pattern to that described in depression, showing reduced FRN for motivational outcome information [42] and dampened error processing [43], [44]. Further, self-reported reward sensitivity, BAS and sensation-seeking are linked to reduced FRN for both reward and punishment [45], [46]. Finally, reduced FRN has also been reported in psychiatric disorders characterised by impulsivity and risk-taking, including alcohol dependence [47], substance abuse [48], attention-deficit hyperactivity disorder [49] and pathological gambling [50]. We have also demonstrated in a delay-discounting paradigm that immediate rewards elicit smaller FRN than delayed rewards, and that this effect is steeper in individuals prone to hypomanic symptoms [51], consistent with elevated impulsivity in clinical samples of BD [1]–[3]. Collectively, evidence suggests that manic symptoms would be associated with a similar FRN attenuation for both reward and punishment.

Here we sought to characterise motivational processing in well-functioning individuals with psychometric vulnerability to BD (but with no psychiatric diagnosis), allowing us to exclude confounds from psychotropic medication and hospitalisation, and to potentially uncover vulnerability markers for the disorder. Because manic and depressive symptoms frequently co-occur in BD (e.g. [52]) and these features are associated with opposing perturbations of FRN and other markers of motivational processes (see above), we excluded depressive vulnerability so as to isolate electrophysiological markers uniquely associated with susceptibility to hypomania. We hypothesised that these individuals would show a bias towards positive evaluation of motivational outcomes and impaired learning of reward contingencies. Given that the FRN codes subjectively advantageous outcomes with reduced amplitude, relative to disadvantageous ones, we predicted 1) reduced FRN amplitude for gain relative to losses, and 2) that the hypomania-prone individuals would show a smaller FRN for gains (relative to the other groups), indicative of a greater hedonic impact of rewards in this group. A second prediction was that FRN deflection elicited by punishment outcomes would also be reduced in the hypomania-prone group (relative to the other groups), consistent with findings that aversive outcome processing is dampened by trait impulsivity.

## Materials and Methods

### Participants

49 right-handed individuals (24 male, 25 female, *M*_age_  = 21.4, *SD*  = 2.41) were sampled from a larger pool (N = 652) of students at the University of Manchester that had completed an online battery of questionnaires (see below). An online screening questionnaire was used to exclude participants reporting current or past history of psychiatric or neurological illness and receiving psychotropic medication.

### Self-report measures

All participants from the larger pool had completed the 48-item Hypomanic Personality Scale [14], 21-item BIS/BAS scales [16], and 24-item Dysfunctional Attitudes Scale; [53]. Both the HPS [14], [15] and BIS/BAS scales [54] have been robustly demonstrated to predict BD, whereas the DAS has been shown to measure depressive cognitive style [55], [56]. Hypomanic and depressive symptoms often co-occur in clinical [57] and non-clinical [58] samples of BD. Hence, in order to isolate differences specifically associated with hypomanic symptoms, participants with depressive cognitive styles were excluded using a DAS cut-off of one standard deviation above the mean (*M* = 98.5, *SD*  = 17.8). Three groups were then selected on the basis of their online HPS scores and contacted to take part in the study. Using established HPS cut-offs (e.g. [59], [60]) we defined high hypomania (Hi-hyp; *n* = 17) by the upper decile of the larger pool (N = 652). A medium hypomania (Mid-hyp; *n* = 15) was defined by scores around the mean (*M* ± *SD*), and a low hypomania group (Lo-hyp; *n* = 17) comprised individuals with HPS scores in the lower two deciles. All groups were selected to have near-equal distribution of male and female participants and did not differ significantly in age [*F(2,42)*  = 3.39, *p* = .715].

### Stimuli and Task

**Figure 1:**
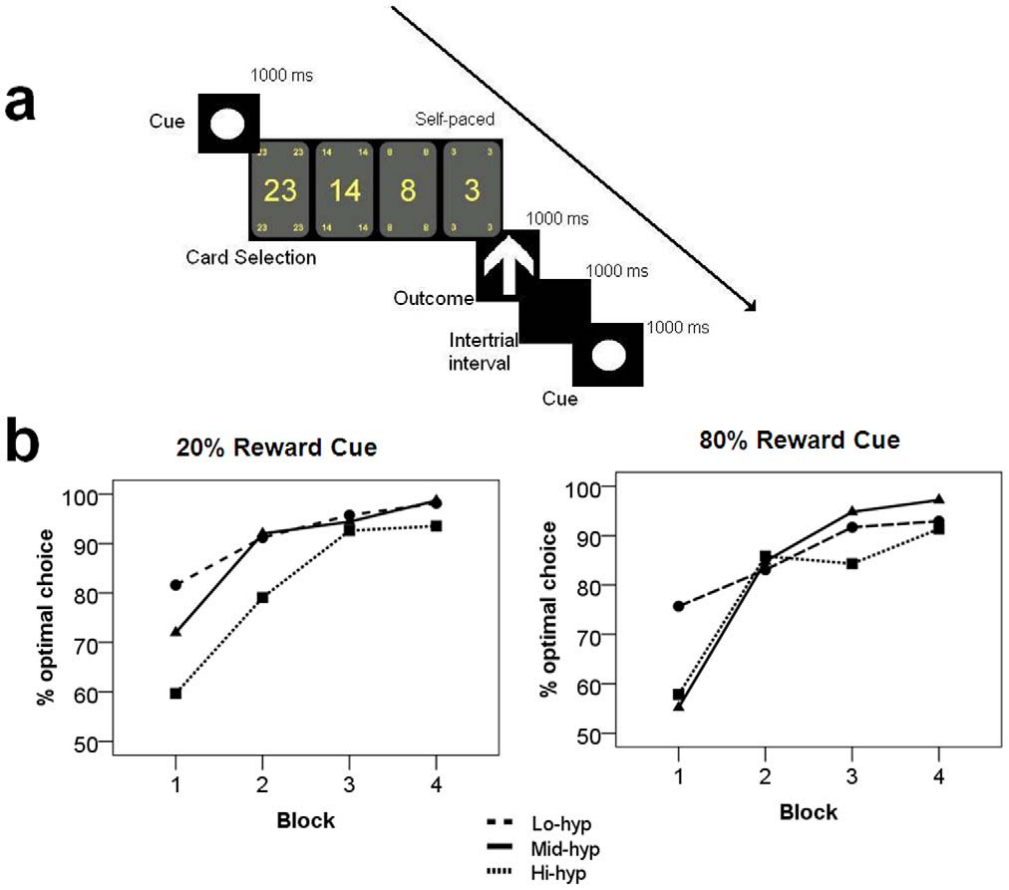
Schematic of experimental design and behavioural data. a) Diagram of a single trial. Participants learned the reward contingencies associated with the three cues (circle, square, triangle) and decide how much to bet (23, 14, 8, or 3 pence). One second later they received feedback indicating gain (up arrow) or loss (down arrow). b) Percentage of optimal choices by group, cue and block. Optimal choices were defined as either of the lower bet sizes (for the 20% reward condition) and either of the larger bet sizes (80% reward condition).

Participants played a computerised card game, in which they learned the reward contingencies of three cues (circle, square, and triangle) associated with 20%, 50% and 80% chance of reward (which are referred to as ‘punishment’, ‘50–50’ and ‘reward’ conditions, respectively). The contingencies carried by each shape were counterbalanced across participants. Participants used this information to guide their choices of how much to bet in pence (23p, 16p, 8p, 3p). These values are in accordance with those routinely reported in the literature [37], [61]–[63] and were piloted, along with the contingencies, to confirm that they elicit reward in the present setting. After placing a bet, feedback was delivered indicating whether the sum of money was won or lost (indicated by an upward or downward arrow respectively). Participants were instructed to maximise their winnings whilst minimising their losses, and that they would be paid their actual winnings at the end of the experiment. See Figure 1a for a schematic diagram of the trials. The experiment consisted of four blocks of 90 trials, with a five minute break after each. Of the 360 total trials, these were equally distributed into the three categories (*i.e*. 120 reward, 120 punishment, 120 50–50 trials) and hence yielded six outcomes with the following frequencies. Reward condition: 96× gain (‘expected gain’), 24× loss (‘unexpected loss’); Punishment condition: 24× gain (‘unexpected gain’), 96× loss (‘expected loss’); 50–50 condition: 60× gain, 60× loss (‘50–50 gain’ and ‘50–50 loss’, respectively).

Unbeknownst to participants, everyone was reimbursed £10 regardless of performance (the average profit made when the paradigm was piloted).

### EEG acquisition, processing and analysis

Continuous EEG recording was obtained from 64 scalp electrodes using ActiveTwo system (BioSemi, Amsterdam, Netherlands) and Actiview® software (BioSemi, Netherlands). Pre-processing was performed off-line using Brain Electrical Source Analysis 5.2 (BESA; Gräfelfing, Germany). Data was re-referenced to the average of all channels and only trials from the second block onwards were analysed, to ensure that participants had learned the reward contingencies. Ocular artefact correction was performed on the entire file using a cut-off of ±150 μV using an established approached [64]. Any outstanding portions of the EEG file with excessive absolute amplitude (>120 μV), voltage gradient between two neighbouring data points (>75 μV) or low signal (<.01 μV). Epochs were defined as −500 ms to 1000 ms relative to the outcome feedback (vertical arrow indicating gain or loss), with baseline defined as the 100 ms prior to feedback. The data were then averaged using a high-pass filter of 0.1 Hz (forward phase shift). MATLAB® 6.5 (MathWorks, USA) was used to pick peaks for our ERPs of interest on averages (see below) filtered with a low-pass filter of 30 Hz. Participants with fewer than 18 trials in each condition were excluded.

The feedback-related negativity (FRN) was identified as a negative deflection in frontal electrodes occurring 250–300 ms post feedback. We measured the FRN as the peak-to-peak difference between the P2 (maximum in the window 150–230 ms) and N2 (minimum in the window 180–320 ms) using an algorithm similar to Holroyd et al [65]. Hence FRN voltage is always a positive value when there is an N2 deflection, and equals zero if there is no negative deflection [65], [66]. This approach controls for the effect of the preceding P2 component on FRN measurement. Supplementary analyses measured the FRN by mean amplitude and difference wave (see Supplementary Materials S1; Figure S2). Analyses were conducted on a frontocentral electrode cluster (F1, Fz, F2, FC1, FC2, and FCz). All participants had at least 16 trials per averaged condition and the mean number of trials for final analysis did not differ between hypomania groups (*p* = .788).

### Statistical Analysis

Task performance was quantified as the percentage of ‘optimal bets’ each participant made (*i.e*. one of the two larger bet sizes for reward trials, or one of the two smaller bet sizes for punishment trials). Participants that did not make these selections on at least 75% of trials in blocks 2, 3 and 4 were presumed to have not learned the reward contingencies and were excluded from further analyses. Proportions of choices were normalised through square-root transformation [67] before using parametric tests. Group differences in task performance and amplitudes on the electrophysiological measures were tested using repeated measures analysis of variance (ANOVA). To dissociate whether the processing of reward and punishment showed a specific relationship with hypomania, we adopted an established approach [45] in which neural responses to reward and punishment were entered into the same step of a regression analysis with HPS score as the outcome variable.

### Ethical Statement

The study was approved by the University of Manchester research ethics committee. Informed written consent was obtained from all participants and the study was conducted in accordance with the Declaration of Helsinki.

## Results

### Personality and symptom questionnaires

In the screening sample (*n* = 652; *M* = 19.9, *SD*  = 8.01), HPS score positively correlated with the BAS subscales: drive (*r* = 0.268, *p*<.001), reward responsiveness (*r* = 0.21, *p*<0.01), and fun-seeking (*r* = 0.415, *p*<.001). These correlations were also present in the final sample recruited into the study (*n* = 49, all *p*<.03) confirming similarities between our sample and clinical populations on these measures. Due to the DAS-24 cut-off, the final groups did not differ on level of depressive symptoms [*F*(2, 44)  = 2.02, *p* = .146], allowing us to selectively examine effects related to susceptibility to hypomania.

### Reward learning task

Four participants (two Lo-hyp and two Hi-hyp) did not show evidence for learning the reward contingencies and were excluded. The final sample was therefore as follows: Lo-hyp (*n* = 15), Mid-hyp (*n* = 15), and Hi-hyp (*n* = 15). All participants included in final analyses (*n* = 45) were able to correctly identify the cues associated with low, medium and high probability of reward when debriefed after the task.

When normalised percentage of optimal bets was entered into a two-way ANOVA with factors: cue (2), block (4) and hypomania group (3), there emerged a main effect of block [*F(3,120)*  = 51.56, *p*<.001]. Contrasts showed that the final sample made significantly more optimal bets in block 2 than in block 1 (*p*<.001), confirming that learning had taken place (Figure 1b; mean bet sizes shown in Figure S1). A block-group interaction [*F(6,120)*  = 3.23, *p* = .006] and a block-cue-group interaction [*F(6,120)*  = 2.58, *p* = .022] also emerged, with a main effect of group approaching significance [*F(2,40)*  = 2.59, *p* = .065]. Contrasts for the block-group interaction confirmed that groups differed by optimal choices in block one, with confidence intervals for the marginal means indicating that Hi-hyp participants made fewer optimal choices in block one than the other groups. The three-way interaction indicated that although Hi-hyp participants showed an increase in optimal choices between block one and two, this increase was steeper for the reward cue than penalty cue, relative to the other groups. A cue-group interaction failed to reach significance for blocks 2–4 (*p*≥.127), however, nor were the effect of group or remaining group interactions significant for these blocks (*p*≥.095), indicating that all groups reached the same levels of performance after block 1.

### Electrophysiological results

**Figure 2:**
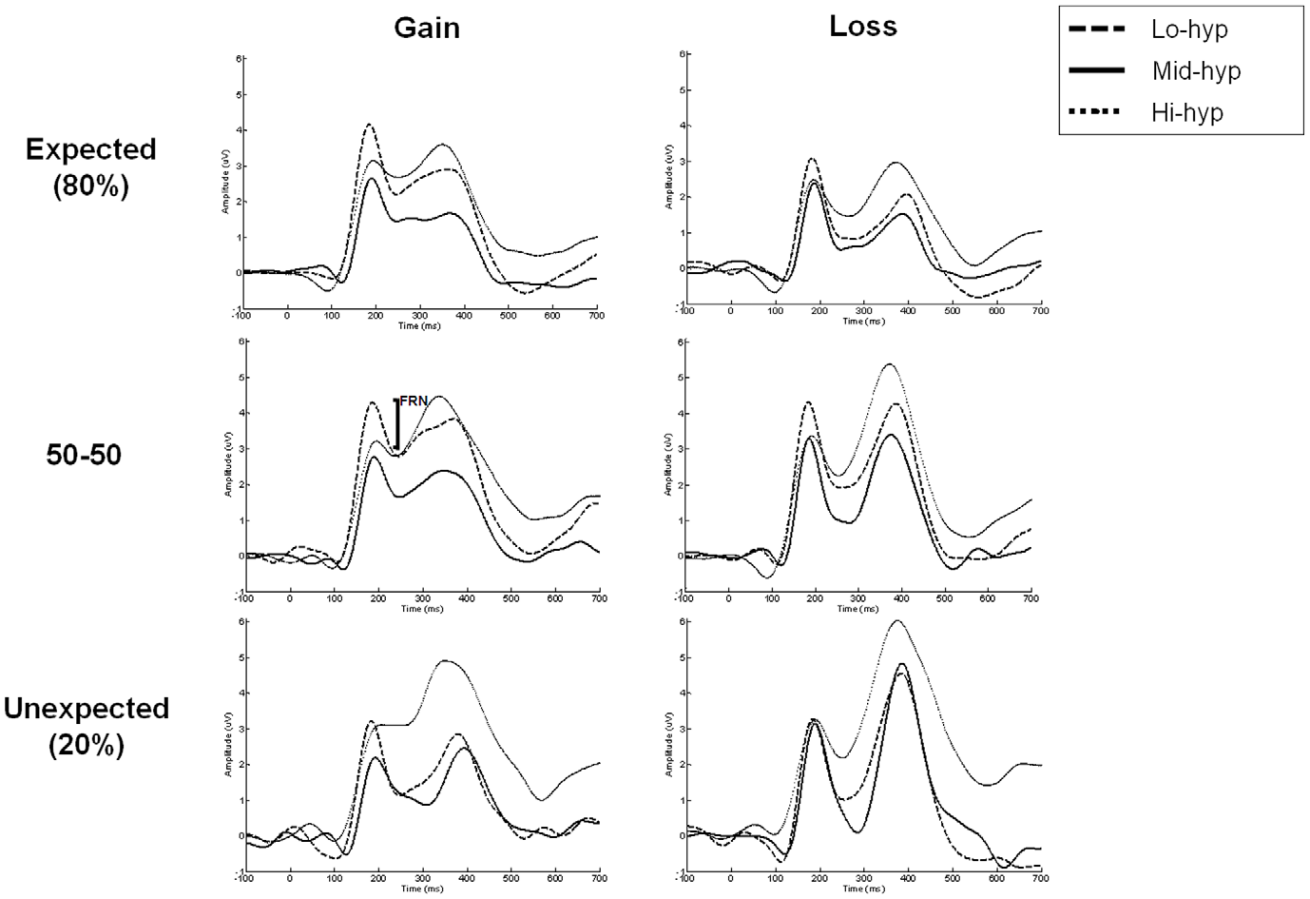
Average waveforms for all conditions by group. Hi-hyp show reduced (**less negative; more gain-like**) **feedback-related negativity compared to the other groups.**

**Figure 3:**
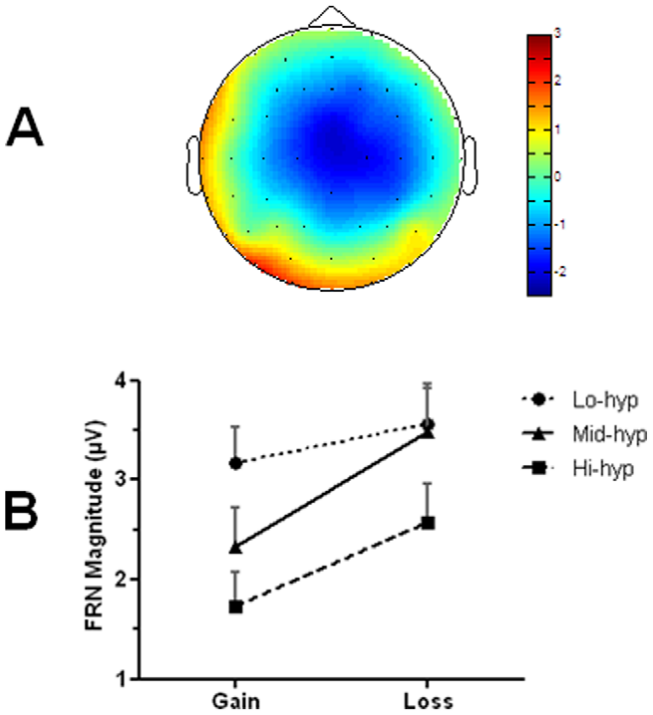
Topography of feedback-related negativity (**FRN**) **and its modulation by group and valence.** a) Topographical plot of the 50/50 difference wave (loss minus gain; 260–320 ms) shows typical frontocentral distribution of FRN. b) FRN magnitudes for reward (gain) and punishment (loss) across groups. The Lo-hyp group show similar FRN morphology to the Mid-hyp group for losses, but larger FRN for rewards (more loss-like) suggesting reduced reward sensitivity. FRN is reduced in the Hi-hyp group for both gains and losses, suggesting that both outcomes are subjectively experienced as more advantageous (*i.e*. more gain-like), relative to the other groups.

Consistent with the literature, the FRN deflection was modulated by both expectancy and outcome valence (Figure 2), and exhibited a frontocentral topography (Figure 3a). An ANOVA was carried out with two within-group factors, cued reward probability (20%, 50%, 80%) and outcome valence (gain, loss), and one between-groups factor: hypomania group (low, mid, high). Main effects of outcome [*F(1,42)* = 40.58, *p*<.001], cue [*F(2,42)* = 4.04, *p*<.03] and hypomania group emerged [*F(2,42)* = 3.24, *p*<.05], as well as an interaction between outcome and hypomania group [*F(2,42)* = 3.71, *p*<.04]. There was a trend for a cue-outcome interaction, but this did not reach significance (*p* = .11). Contrasts across all participants confirmed that the FRN was larger both for losses (relative to gains), and for unexpected outcomes (relative to expected: 20% vs. 80%; *p*<.02), confirming that the task was appropriate for measuring neural responsiveness to reward and punishment.

Between-groups contrasts for the main effect of hypomania group showed that the FRN was significantly reduced in the Hi-hyp relative to Lo-hyp group (*p*<.02). This confirmed that the Hi-hyp group produced smaller FRNs across task conditions (Figure 3b). The outcome-by-group interaction was explored using separate ANOVAs for each group. Whilst a valence effect was significant in the Mid-hyp and Hi-hyp groups (p≤.001), a trend for reduction in this effect in the Lo-hyp group (Figure 3b) did not reach statistical significance in the Lo-hyp group (p = .081). The main effect of group also remained significant [*F(1,43)* = 4.02, *p*<.05] when a median split was used to divide the sample into two larger hypomania groups: low (*n* = 23) and high (*n* = 22).

When the 50% outcomes were entered into a repeated measures ANOVA (factors: outcome and group), main effects of outcome [*F(1,42)* = 18.5, *p*<.001] and group [*F(1,42)* = 2.71, *p* = 07] were again found to be significant or approach significance. To further specify the relationship between hypomania and motivational processing, FRN amplitudes for gain and loss outcomes were entered as predictors of HPS score in the same step of a regression analysis. Outcomes from the 50–50 condition were selected because of equivalent reward probability and magnitude. The resulting model accounted for 14% of the variance [*F*(2,42) = 3.424, *p* = .042]. Whereas the gain FRN accounted for a significant amount of this variance (p = .031), the loss FRN did not (p = .749), suggesting that vulnerability to hypomania is particularly associated with neural sensitivity to reward outcomes.

## Discussion

In this study, we identify differences in the neural processing of motivational information in individuals vulnerable to hypomania. The results provide further electrophysiological evidence linking reward system alterations to risk-taking and impaired learning in BD.

The hypomania-prone (Hi-hyp) group showed impaired learning of the reward contingencies in the first block, making significantly fewer optimal choices than the other groups and accruing the lowest task earnings. These results are in agreement with decision-making and learning impairments reported in clinical populations [68], [69]. Poor performance in the punishment condition may also indicate greater risk-taking predilection (*i.e* placing large bets in spite of the odds). Indeed BD is associated with risk-taking clinically (DSM-IV-TR; [4]), perhaps due to reduced sensitivity to modulatory psychological factors when making risky choices (see [69]). Although we did not collect explicit measures of impulsivity in this study, susceptibility to hypomania was also associated with increased self-report of subjective reward responsiveness and novelty-seeking behaviours (BAS subscales; [16]).

Across all participants, FRN was modulated by outcome valence, appearing larger for losses regardless of how it was measured. This is consistent with previous findings and the view that this component represents the activity of a system evaluating the motivational significance of outcomes (e.g. [66]).

In the main FRN analysis the low hypomania group showed reduced neural differentiation of gains and losses, relative to the mid hypomania group. This was driven by increased (*i.e*. more loss-like) FRN for gains, a finding that has also been reported in a sample exhibiting depressive symptoms [37], and is consistent with a reduced reward response. The hypomania-prone group showed reduced FRN for both outcomes, indicating a tendency to experience both outcomes as more favourable than the other groups (a positive evaluation bias). This effect was particularly pronounced for rewards, consistent with recent electrophysiological evidence of hypersensitivity to immediate reward during a delay discounting task [51] and clinical accounts that mania is related to reward hypersensitivity [6], [7]. In addition, the present finding of reduced FRN for losses fits with the reduced punishment sensitivity hypothesis of impulsivity disorders [41] and may help to explain the detrimental behaviours seen clinically in BD, such as unrestrained spending sprees, substance use, unprotected sex and impulsive suicide attempts (DSM-IV-TR; [4]).

Because bet size varied systematically for two of three cues (participants chose smaller bets in the 20% compared to 80% reward condition, confirming learning of the contingencies), FRN differences may also reflect magnitude. Indeed some studies have found that FRN is sensitive to magnitude, particularly for gains (e.g. [70]), whereas others find it is not (e.g. [71]), so we cannot confidently conclude whether the FRN reduction observed in the hypomania-prone group is driven more by expectancy or magnitude. However, group differences in outcomes following the 50% cue, which were equivalent with respect to magnitude, suggest that it is reward expectation that deviates in hypomania. This fits with findings that mania is associated with impaired orbitofrontal representation of expected value – and not magnitude [72], and with clinical accounts of grossly increased confidence that goals will be attainable and have favourable outcomes [73].

Our results are also consistent with models of risk-taking as arising from an imbalance between striatal activation and ACC control [45], [74]. Indeed the ACC, a major generator of the FRN [61], is implicated in both affective processing and performance monitoring [75] and shows abnormal activation in depression [76] and mania [77].

Under reinforcement learning accounts of the FRN, the present findings indicate altered prediction error signalling in groups exhibiting either extremely low or high hypomania traits. This is consistent with neuroimaging evidence of altered striatal prediction error signalling in clinical [24] and analogue [26] samples of mania. In hypomania-prone individuals, reduced FRN for gains and losses implies increased (more positive) prediction error activity (see [28]). A similar evaluation bias has been reported in other clinical populations exhibiting impulsive and risky behaviours (e.g. Parkinson's with impulse control disorders; [27]) and in healthy individuals following administration of a DA-enhancing agent [25]. In both of these cases it has been suggested that increased positive prediction errors may induce a persistent “better than expected” evaluation, leading to a greater impact of rewards and a reduced impact of punishment (see [27]). This may drive an expectancy bias towards positive outcomes, as we have demonstrated in a separate neuroimaging study of reward learning in hypomania [26]. Hence learning deficits and repeated risk-taking may both arise from inappropriate reinforcement learning signals that fail to update future expectations. This pervasive “rose-tinted” evaluatory bias parallels neuroimaging evidence that trait unrealistic optimism is maintained by a selective failure to update future estimations in the light of undesirable information [78]. An alternative interpretation of group differences in the FRN when viewed as indexing a prediction error [28], is that they are driven by differences in estimation of the expected value of upcoming outcomes rather than evaluation (post-outcome). The two stages of processing are inextricably linked (prediction error updates future expected value) and so cannot be differentiated by the current design. Indeed this represents a conceptual limitation of FRN studies in general.

A strength of this study is that it examined the relationship between hypomanic symptoms and motivational processing whilst avoiding confounds from depressive symptoms, medication, hospitalisation or comorbidity. However, an intrinsic limitation of this approach is that the sample may not fully represent the range of psychopathology seen in clinical populations (although see [51], which found a neural bias for immediate rewards in a hypomania-prone sample where depressive symptoms were not excluded). Additionally, whilst we cannot rule out that generalised reduction of the FRN in the hypomania-prone group may be due to reduced task engagement, the elevated traits of drive and responsiveness to reward exhibited by this population [73] argues against this interpretation. Our paradigm used free choice to examine risk-taking and, as such, was unable to orthogonalise reward probability and magnitude in all conditions. Also, a relatively low number of unexpected outcome trials were obtained (because they are intrinsically rare in realistic probabilistic tasks). A recent paper advised 20 trials for robust measurement of FRN [79] – 2 more than in the present study. However, the pattern of results did not differ for the 50–50 gain and loss outcomes, which had the same probability and magnitude as each other, and an ample number of trials to satisfy this criterion. Finally, the positivity preceding the FRN showed some task modulation (consistent with previous findings; [80]), which likely accounts for the discrepant findings between the peak-to-peak and difference wave measurement of the FRN. Nevertheless this discrepancy is a limitation of the study and warrants replication with, for example, a less complex task not involving learning or free choice on bet size (see above).

In conclusion, we report differences in the neural processing of motivational information in individuals vulnerable to hypomania. The present findings are consistent with accounts that BD is associated with reward dysregulation [7], [73] and highlight a common neural mechanism contributing to risk-taking and impaired reward learning. A positive evaluation bias may also explain the elevated motivation, confidence, and goal-striving associated with mania [73]. In addition, our findings here and elsewhere [26], [81] demonstrate biological vulnerability markers for BD. These may ultimately lead to more quantifiable risk estimates [82], facilitating early detection and intervention. Our data suggest that appraisal and reflective consolidation of risky events may be a helpful therapeutic approach.

## Supporting Information

Figure S1 **Mean bet size shown by block and group.** Participants alter their bet size after learning the 20% and 80% reward contingencies. Hi-hyp are slower to adjust their bet size in the 20% reward (punishment) condition, consistent with slower learning.
(TIF)

Figure S2 **Probability-group interaction for mean amplitude analysis** (**260–340 ms**)**.** The Hi-hyp group show smaller feedback-related negativity (more positive voltage) in for all outcomes and additionally deviate from the other groups in their processing of unexpected outcomes, showing smaller FRN.
(TIF)

Supplementary Materials S1 (DOC)
