# Supplementary figures and images for: Better Than I Thought: Positive Evaluation Bias in Hypomania
Source: PLoS One. 2012 Oct 17;7(10):e47754. doi: 10.1371/journal.pone.0047754 (PMC3474792; doi:10.1371/journal.pone.0047754)

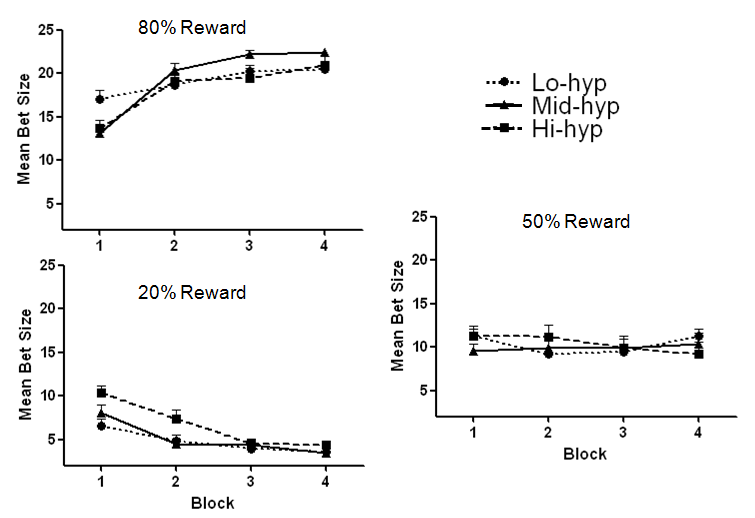

Supplement: Figure S1 — Mean bet size shown by block and group. Participants alter their bet size after learning the 20% and 80% reward contingencies. Hi-hyp are slower to adjust their bet size in the 20% reward (punishment) condition, consistent with slower learning. (TIF) [file pone.0047754.s001.tif]

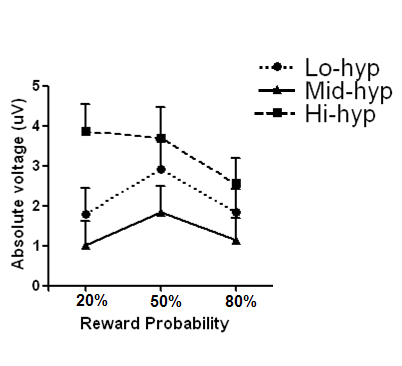

Supplement: Figure S2 — Probability-group interaction for mean amplitude analysis (260–340 ms). The Hi-hyp group show smaller feedback-related negativity (more positive voltage) in for all outcomes and additionally deviate from the other groups in their processing of unexpected outcomes, showing smaller FRN. (TIF) [file pone.0047754.s002.tif]
