# Supplementary material for: Better Than I Thought: Positive Evaluation Bias in Hypomania
Source: PLoS One. 2012 Oct 17;7(10):e47754. doi: 10.1371/journal.pone.0047754 (PMC3474792; doi:10.1371/journal.pone.0047754)
Supplement: Supplementary Materials S1 — (DOC) [file pone.0047754.s003.doc]

**Supplementary Materials**

**Results**

*Difference Wave analyses:*

Difference waves were calculated for each cue (loss-gain waveform), and the minimum voltage occurring 200-400 *ms* was entered into an ANOVA with factors cue and group. The same electrode cluster was used as the peak-to-peak measurement. There were no main effects or interactions (*p*≥.466), indicating that FRN was not sensitive to probability or group differences when measured this way in the present study.

*Mean amplitude analyses:*

The above ANOVA was repeated with FRN values computed using a mean amplitude measurement over the same frontocentral electrode cluster (260-340 ms). Main effects of valence [*F*(1, 42) = 15.7, *p*<.001] and cue [*F* (2, 84) = 8.73, *p*<.001] emerged, as well as a valence-by-cue interaction [*F* (2, 42) = 7.84, *p*=.015]. The main effect of group approached significance [*F* (2, 42) = 2.76, *p*=.075] as did a group-cue interaction [*F* (4, 84) = 2.2, *p*=.075]. In contrast with the peak-to-peak measurement of FRN, there was no outcome-group interaction (*p*=.68).

As with the peak-to-peak analysis, losses elicited larger (more negative) FRN than gains. However, contrasts showed that expected outcomes elicited larger FRN than unexpected (*p*=.046; although see interpretation of group-cue interaction below) or 50-50 outcomes (*p<*.001), and that 50-50 outcomes elicited smaller FRN than unexpected outcomes (*p*=.053). Separate repeated measures ANOVAs exploring the cue-outcome interaction showed that whilst the valence effect was present for 80% (*p<*.001) and 50% (*p*=.003) outcomes, it was absent for the 50-50 condition (*p*=.991). Contrasts performed on the trend for main effect of group showed that the Hi-hyp group had smaller FRN than the Mid-hyp (*p*=.024) but not Lo-hyp (*p*=.19) group. Inspection of the cue-group interaction showed groups differed in their responsivity to unexpected outcomes, with Hi-hyp showing reduced FRN (more positive voltage) rather than greater negativity (see Figure S2). Response in the Hi-hyp group to unexpected outcomes is likely driving the probability effect reported above, as the other groups show equivalent response for expected and unexpected outcomes.

**Supplementary Discussion**

The pattern of FRN modulation by valence was consistent with the literature regardless of measurement approach. Modulation by probability was consistent with the literature when measured peak-to-peak (larger signal for unexpected than expected outcomes), but the opposite pattern was observed for mean amplitude measurement. Mean amplitude measurement also failed to detect an effect of valence for 50-50 outcomes, which is at odds with the peak-to-peak findings and the wider literature. This demonstrates how mean amplitude measurements can be affected the amplitude of the immediately preceding component (in this case, P2) if this component also shows modulation by task parameters (see Figure 3). Peak-to-peak measurement of FRN avoids this confound, but can lead to an underestimation for instances when the P2 is not followed by a negative deflection [1], such as when a temporally contingent positivity is superimposed on the FRN [see 2]. Definition of FRN as a difference wave between gains and losses accounts for these problems but reduces the specificity of this measurement, allowing differences to be driven by any combination of several frontocentral components (*e.g.* P2, N2 and late positivity). More crucially, it also prevents reward and punishment processing to be quantified separately, which is of clear interest clinically. Modulation of the P2 by task parameters was not assessed in this study, but has been reported elsewhere [3] and will be an important avenue for further research.

**References**

1. Wu Y, Zhou X (2009) The P300 and reward valence, magnitude, and expectancy in outcome evaluation. Brain Research 1286: 114-122.

2. Holroyd CB, Pakzad-Vaezi KL, Krigolson OE (2008) The feedback correct-related positivity: sensitivity of the event-related brain potential to unexpected positive feedback. Psychophysiology 45: 688-697.

3. Oberg SAK, Christie GJ, Tata MS (2011) Problem gamblers exhibit reward hypersensitivity in medial frontal cortex during gambling. Neuropsychologia.
